# Ethical approval of studies involving humans and other animals

**DOI:** 10.1017/awf.2025.5

**Published:** 2025-02-12

**Authors:** Birte L. Nielsen, T. Bas Rodenburg

**Affiliations:** 1 Universities Federation for Animal Welfare, Hertfordshire, UK; 2 Department Population Health Sciences, Utrecht University, The Netherlands; 3 Editors-in-Chief of Animal Welfare

**Keywords:** animal welfare, ethical review, publishing, journal, harm-benefit, ethical implications

The ethical approval of scientific studies involving animals has come a long way in recent years. No, it is not yet perfect, and there are still issues to resolve but, overall, the processes of ethical assessment (appear to) have improved across animal welfare science as a whole. We realise that it can be dangerous to make general statements like that, as they almost send out an invitation to demonstrate where the current system is not working optimally. Indeed, one of us recently co-authored an article to highlight some of these issues (Olsson *et al.* 2022). This editorial will instead give a brief description of what we have tried to do with *this* journal in terms of moving ethical considerations of animal research forward. It is a work-in-progress, but by explaining the policy of the journal here, we hope to inspire researchers and article authors to take some of our suggestions to heart.

Ethical considerations are at the forefront of all animal research (see ASAB Ethical Committee/ABS Animal Care Committee 2023 for guidelines for the ethical treatment of nonhuman animals in behavioural research and teaching). This is also the case for *Animal Welfare*, and it is reflected in our format-neutral submission guide: We ask for only four things to be ensured prior to submission, and one is that the manuscript contains information on ethical considerations associated with the study/work (in case you were wondering, the other three are easily readable layout, continuous line-numbering, and a section on Animal Welfare Implications of the study/work).

Commonly in a scientific article, the information on the ethical approval of a study involving animals consists of the name of the Animal Ethics Committee (AEC), Institutional Animal Care and Use Committee (IACUC), or Animal Welfare and Ethical Review Body (AWERB), together with the approval document reference ID. Although these suffice to demonstrate that ethical evaluation and approval of the protocol has taken place, it doesn’t inform the reader of what was done to ensure or mitigate the welfare of the animals involved. Some authors include information on the 3Rs (Replacement, Reduction, and Refinement), which is very useful (and pleasing for us, as William Russell and Rex Burch [1959] developed these while they were UFAW Scholars in the 1950s). The essence of an ethical review, however, is the **harm-benefit analysis**, where the potential benefits of an experiment are weighed against the likely harms caused to the individuals involved.

We therefore ask the authors submitting their manuscripts for publication in *Animal Welfare* to describe in more detail the ethical considerations they had (the harm-benefit weighing) and what they did to mitigate the welfare impact on their subjects (the 3Rs). The wording from the section on Ethical Considerations (taken from the Author Instructions for the journal) is repeated below:

“*Where ethical considerations arise (e.g. if procedures compromise animal welfare or give rise to other ethical concerns, such as when human subjects are involved), these must be addressed in the methods section. Any ethical implications and justifications of the experimental design or procedures should be described; details should be provided of licences or other permissions required for the work from ethical review bodies. If ethical approval was not required to carry out the study, this should be made explicit, and a detailed justification given in the methods section. Measures undertaken to minimise the adverse welfare impact on animals involved, including choice of sample size, use of pilot tests and predetermined rules for intervention, should be described. The fate of all animals used in the study should be detailed. Steps taken to enhance the welfare of animals involved (e.g. through environmental enrichment) should also be outlined.*” (from the online Author Instructions for *Animal Welfare*)

All authors are thus encouraged to include a more detailed description of their animal welfare and ethical considerations – also when the study had ethical approval. This has the potential to inform and educate readers and fellow scientists on ways to improve the lives of animals when they take part in research. Not all articles published in *Animal Welfare* have expanded ethical considerations – yet. But the journal editors will keep encouraging it and, rather than making it compulsory, we are hoping that reminders – like this editorial – can lead to more and more articles including slightly more information than just a committee name and approval number. It is worth mentioning here that, based on assessment by the Editorial Board, the journal has rejected (and will continue to reject) papers, including studies where an ethical review committee has given approval, if the harm-benefit assessment is not clear, and where the question asked or the protocol used did not justify the involvement of animals. Our aim is that articles involving humans and other animals published in *Animal Welfare* are based on results obtained using experimental protocols that take their outset in the welfare of the subjects studied, and with ethical considerations at the forefront of the researchers’ minds. So, if you are thinking about submitting a manuscript to *Animal Welfare*, please include a few sentences on the ethical considerations you had before carrying out your study.
